# Damage Classification Using Supervised Self-Organizing Maps in Structural Health Monitoring

**DOI:** 10.3390/s22041484

**Published:** 2022-02-15

**Authors:** Gilbert A. Angulo-Saucedo, Jersson X. Leon-Medina, Wilman Alonso Pineda-Muñoz, Miguel Angel Torres-Arredondo, Diego A. Tibaduiza

**Affiliations:** 1 Department of Electrical and Electronic Engineering, Universidad Nacional de Colombia-Sede Bogotá, Cra 45 No. 26-85, Bogotá 111321, Colombia; gaangulosa@unal.edu.co (G.A.A.-S.); dtibaduizab@unal.edu.co (D.A.T.); 2 Control, Modeling, Identification and Applications (CoDAlab), Department of Mathematics, Escola d’Enginyeria de Barcelona Est (EEBE), Campus Diagonal-Besòs (CDB), Universitat Politècnica de Catalunya (UPC), Eduard Maristany 16, 08019 Barcelona, Spain; 3 Department of Mechatronics Engineering, Universidad de San Buenaventura Sede Bogotá, Carrera 8H No. 172-20, Bogotá 111156, Colombia; 4 GENTE Group, Department of Electromechanical Engineering, Universidad Pedagógica y Tecnológica de Colombia, Tunja 150462, Colombia; wilman.pineda@uptc.edu.co; 5 MAN Energy Solutions SE, 86153 Ausburg, Germany; miguel.torres@man-es.com

**Keywords:** structural health monitoring, machine learning, self-organizing maps, damage classification, principal component analysis, piezoelectric, data acquisition system

## Abstract

Improvements in computing capacity have allowed computers today to execute increasingly complex tasks. One of the main benefits of these improvements is the possibility of developing machine learning algorithms, of which the fields of application are extensive and varied. However, an area in which this type of algorithms acquires an increasing relevance is structural health monitoring (SHM), where inspection strategies and guided wave-based approaches make the evaluation of the structural conditions of an aircraft, vessel or building among others possible, by detecting and classifying existing damages. The use of sensors, data acquisition systems (DAQ) and computation has also allowed these damage detection and classification tasks to be carried out automatically. Despite today’s advances, it is still necessary to continue with the development of more robust, reliable, and low-cost structural health monitoring systems. For this reason, this work contemplates three key points: (i) the configuration of a data acquisition system for signal gathering from an an active piezoelectric (PZT) sensor network; (ii) the development of a damage classification methodology based on signal processing techniques (normalization and PCA), from which the models that describe the structural conditions of the plate are built; and (iii) the use of machine learning algorithms, more specifically, three variants of the self-organizing maps called CPANN (counterpropagation artificial neural network), SKN (supervised Kohonen) and XYF (X–Y fused Kohonen). The data obtained allowed one to carry out an experimental validation of the damage classification methodology, to determine the presence of damages in two aluminum plates of different sizes, where masses were added to change the vibrational responses captured by the sensor network and a composite (CFRP) plate with real damages, such as delamination and cracks. This classification methodology allowed one to obtain excellent results by validating the usefulness of the SKN and XYF networks in damage classification tasks, showing overall accuracies of 73.75% and 72.5%, respectively, according to the cross-validation process. These percentages are higher than those obtained in comparison with other neural networks such as: kNN, discriminant analysis, classification trees, partial least square discriminant analysis, and backpropagation neural networks, when the cross-validation process was applied.

## 1. Introduction

The need to ensure the proper performance of the structures during its operation under different conditions, including environmental and operational conditions, has allowed the development of new areas and strategies for monitoring these structures and ensures the reliability of its use by reducing the risk during its operation. This problem has been tackled by means of structural health monitoring (SHM), where non-destructive testing becomes an integral part of the structure by using permanently installed sensors, which allow one to find out the current health of the structure via the analysis of the information from the sensors. In general, SHM facilitates the analysis of the structures from two points of view, the first by the model construction, which requires good knowledge of the structure and the use of complex models to model its behavior, and detect abnormalities that can be considered as a damage. The second point of view is oriented to the use of data-driven algorithms where data from the sensors provide all the information to identify the damage. According to Rytter [1], the levels of damage diagnosis cover damage detection, damage localization, the identification of the type of damage, the calculation of the extent of damage, the prognosis of the remaining lifetime, and the design of smart structures with self-evaluating, self-healing, or control capabilities. From the point of view of data-driven algorithms, the use of pattern recognition is the most used, because of the possibilities of comparison with the structure in a healthy state, and detecting different patterns to define the presence of damages, and its location, among others [2,3]. Different methods are used to collect the information from the structure, however, sensor networks that collect the dynamic response generated by actuators or external inputs are the most used. The basic idea is that changes in the physical properties of the structure due to a damage will cause changes that can be detectable by the data-driven algorithms [4].

SHM has experienced significant advances in recent decades, motivated by the persistent need to improve data acquisition systems and methodologies for detecting, classifying, locating, and quantifying damage, all with the aim of obtaining increasingly reliable results during the diagnosis of a structure [5,6]. The objective of the SHM is to detect structural changes in a timely manner, safeguarding the safety of people, reducing maintenance time and costs, which tend to be high when conventional diagnostic methodologies such as visual inspection, X-rays and thermographic images are carried out [5].

Due to the growing interest in contributing to the area of structural health monitoring (SHM), there remains a need to continue the research and development of damage classification systems [7,8,9]. More specifically, hardware used in data acquisition and excitation, such as sensors (piezoelectric, for example), is needed, since they are elements that are in direct contact with the structure, and therefore equipment must be able to provide reliable data [2,10,11]. On the other hand, advances in computing have recently allowed the development and implementation of machine learning algorithms to model the biological system of human neurons, in order to solve complex problems, perform predictive analyses, and carry out classification tasks, which are the goals of this article [5,12]. However, structural monitoring systems based on ultrasound inspection techniques with piezoelectric transducers require a series of complementary equipment that allows carrying out structural evaluations in the most efficient and automated possible way [13,14]. One of these complementary equipments is the multiplexing system of signals [15]. As already mentioned, a structure can be instrumented with the network of piezoelectric transducers; its function is to produce a mechanical wave that propagates throughout the structure and is then captured and analyzed to verify the existence of structural anomalies [5,16]. When this structure is not enough to use a single piezoelectric as an actuator, it is necessary to alternate that actuator function to ensure that the entire structure has been inspected. One option to carry out this task is in an automated way through a multiplexing system [17].

When carrying out each experiment, a large amount of data is collected for each sensor, which is why it is necessary to implement methodologies that allow all the information to be processed, and reduce the data sets, to make the damage classification tasks more efficient, such as machine learning. Today, machine learning algorithms facilitate the tasks of data processing, pattern recognition, and classification, among others, being indispensable tools in many areas of study [13,14]. These machine learning algorithms are classified into three groups: supervised, semi-supervised, and unsupervised. The first group refers to those algorithms of which the learning is determined by prior information, on which it is based on perform groupings within a data set, contrary to unsupervised algorithms that are capable of autonomously performing classification tasks without any prior information. A third group called semi-supervised algorithms combine supervised and unsupervised algorithms into one [18,19,20].

Numerous works have been carried out in the area of structural health monitoring, proposing different procedures with which they ensure obtaining more reliable and precise results in terms of the diagnosis of a structure. For damage classification in SHM, methodologies based on machine learning algorithms contemplate the use of algorithms such as k-means and k-means++ [21,22,23], other works explore bio-inspired algorithms such as AIS (artificial immune system) [24,25], and even combine this type of algorithms with other neural networks [26,27]; others explore and compare decision trees, support vector machines (SVM), and k-nearest neighbors (kNN) [14,28,29,30]. Other studied algorithms in SHM are: naive Bayes, feed forward networks (FNN) [31], Bayesian ANN [32], probabilistic neural networks (PNN) [33], feed-forward backpropagation (FFBP) [34], and general regression neural networks [35], to name the most novel and studied. In all this literature, it is possible to find experimental results where different environmental conditions are involved, and various types of structures and even different normalization techniques are implemented.

In this article, supervised and semi-supervised algorithms are studied, and the effectiveness of the damage classification process is evaluated in two aluminum plates instrumented with four piezoelectric transducers. The algorithms studied are based on self-organizing maps, which is a machine learning technique based on neural networks where each neuron is related to the others according to a similarity metric calculated from the analysis of incoming data. The different groupings of neurons that can exist indicate the amount of structural states present [36,37,38]. The algorithms implemented were three neural networks based on SOM (self-organizing maps) [39]: CPANN (counterpropagation artificial neural network) [40,41], SKN (supervised Kohonen network) [42] and XYF [42] (X–Y fused Kohonen).

Self-organizing maps have been widely studied in the SHM area for damage classification tasks in structures with different characteristics [19,43,44]. However, despite the developments that exist in classification methodologies based on this type of algorithms, it is necessary to continue with the study and development of new methodologies. For this reason, this paper proposes a classification methodology with self-organizing maps based on supervised and semisupervised learning artificial neural networks mentioned above (CPANN, SKN, and XYF). The performance of these neural networks in damage classification tasks in SHM has not been explored in depth. In literature, only a few works explore the CPANN network in damage detection tasks, with satisfactory results [45,46].

The result of this work was a fully functional structural monitoring system, composed of an active piezoelectric sensor network, a data acquisition system (DAQ) capable of generating the excitation signal, and collect all the data from the structure under inspection, and a classification methodology based on self-organizing maps. This system stands out for its simplicity, versatility, easy implementation and low cost, since most of its physical structure (oscilloscope and sensors/actuators) is generic. This work takes, as a reference, the work in [47], and gives news perspectives by adding new pre-processing and classification methods, as explained before.

This article is organized as follows. Section 2 describes the elements that comprise a structural monitoring system, including the functions of each one. In Section 3, a brief description of the concepts involved in this study is given, covering the physical phenomena, pre-processing techniques, and classification algorithms implemented. Section 4 describes the classification methodology developed and Section 5 presents the results obtained.

## 2. Structural Health Monitoring System

The typical configuration of a structural health monitoring system is composed of the following elements: sensor/actuator network, multiplexers, data acquisition system, and a damage identification methodology. The tasks of a system that uses piezoelectrics as an active sensor network are listed below,

- Excitation of a network of piezoelectric transducers by means of a signal previously programmed in the arbitrary signal generator.
- Signal multiplexing capacity.
- Collection, conditioning and storage of data obtained from the piezoelectric sensor network.
- Data analysis for the classification of damage in the analyzed structure using self-organizing maps.

Based on this description, the elements of a structural monitoring system are detailed in the following subsections.

### 2.1. Sensors

The use of piezoeletric sensors is very common in the area of structural health monitoring [10,48,49]. Its ability to work as a sensor and as an actuator avoids the use of elements that could only be dedicated to the generation of mechanical waves, as well as to the use of sensors that are only limited to detecting the response wave, thus allowing a reduction in costs. On the other hand, its low cost due to the simplicity in its construction makes piezoelectric elements ideal when the intention is to seek economy. Additionally, the results obtained in its implementation throughout numerous experiments have been quite satisfactory in the tasks of detection, classification, localization and the quantification of damage in structures that could be metallic or made of composite material [17,50]. These tasks can be carried out with this type of sensors, even when the structure is subjected to temperature variations [4].

### 2.2. Multiplexer

**Figure 1:**
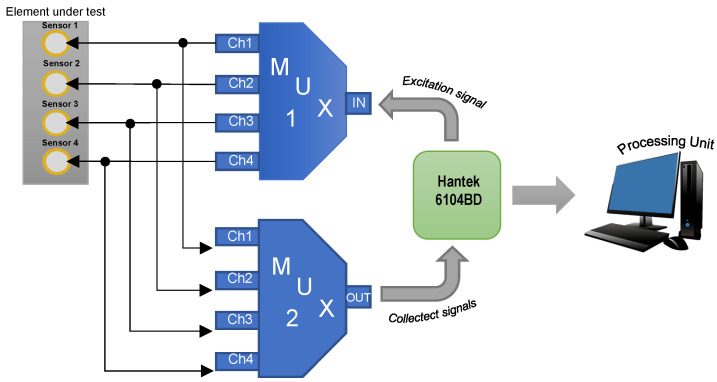
Multiplexing system scheme.

For the control of the output signal of arbitrary waveform generator and input signals to the data acquisition system, it is necessary to implement a multiplexing system that allows conducting the excitation signal to the corresponding sensor, and enables the input channels through which the signals obtained from the piezoelectric sensor network are collected. A simplified scheme of this system is shown in Figure 1. It is valid to clarify that an actuation phase consists of assigning a sensor from the network as the actuator, that is, the element in charge of generating the wave that propagates through the plate, and in the same way, assigning the piezoelectrics that act as a sensor, which captures the mechanical wave and transforms it into an electrical signal. The multiplexing system described in Figure 1 has a simple construction based on an Arduino board, and an 8-channel relay module (4 for the control of the excitation signal and 4 for the control of the channels of data acquisition system). The main task of the Arduino board is to control the channels of the relay module by means of logic signals, according to the actuation phase.

### 2.3. Data Acquisition System (DAQ)

A data acquisition system is an essential element in structural monitoring tasks, therefore, its selection must be careful, so that the collected data can be later processed and interpreted using machine learning algorithms. This work sought to implement a system that incorporated an oscilloscope and an arbitrary signal generator in a single device. As an additional requirement, this generator had to be programmable, since the type of signal required for the excitation of the sensors is not configured by default in any equipment for generic use. This equipment also had some additional characteristics to those already mentioned, such as channels for data acquisition, an adjustable output frequency within the ultrasound range, from 20 kHz onwards, however, the investigations carried out in SHM allowed establishing a frequency limit of 1 MHz [51]. From a detailed review, a device with these characteristics is the Hantek^®^ 6104BD, which has four channels for data acquisition, with a resolution of 8 bits.

### 2.4. Software and Communication

**Figure 2:**
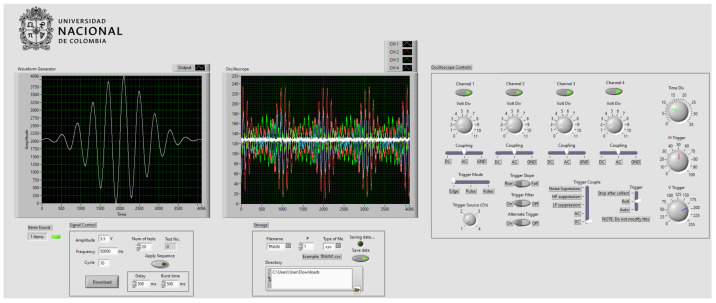
Graphical interface of the Hantek 6104BD control software.

**Figure 3:**
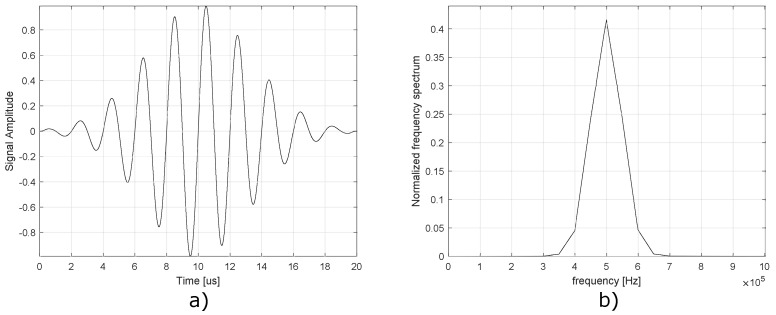
Excitation signal applied to PZT transducer in each actuation phase. (**a**) Time domain. (**b**) Frequency domain.

The control and management of the operations for Hantek^®^ 6104BD equipment require the development of software which can be elaborated in three different development environments indicated by the equipment manufacturer: LabVIEW^™^, Visual C++, and Visual Basic. The LabVIEW^™^ [52] environment was selected for several reasons. First, it is a graphical environment, in which the entire process of the program is structured from blocks that represent a specific function. This feature makes it more intuitive, easy, and quick to understand, compared with other types of environments, in which the program is built from commands of a programming language, of which the learning can take a long time. In the same way, LabVIEW^™^ allows one to build graphical interfaces easily, and there is a lot of information available on the Internet, such as tutorials, guides, etc., Figure 2 illustrates the graphical interface of the designed control software. An important functionality in LabVIEW^™^ is the possibility of using MATLAB^®^ commands, which make it possible to build the required signal through code, since it elaborates that this signal using LabVIEW^™^ functions through blocks is a cumbersome task. Piezoelectric transducers are excited with a signal called tone burst, and it is elaborated by trigonometric functions and normal distribution functions to obtain a waveform as the one shown in Figure 3. The reason for using tone burst type signals in SHM is because, in a reduced bandwidth, it is capable of gathering a series of oscillations, preventing the collected signal from containing too many harmonics that make it difficult to detect structural anomalies [53]. The signal characteristics are: 7 V peak-to-peak and a frequency of 500 kHz. The sampling frequency has been set to 50 MHz. To define this frequency, the Nyquist–Shannon sampling theorem [54] has been considered. This theorem establishes that the sampling frequency must be at least twice the frequency of the measured input signal, that is, ($f_{sample}\geq 2f_{signal}$), to guarantee an adequate reconstruction of the collected signal.

**Figure 4:**
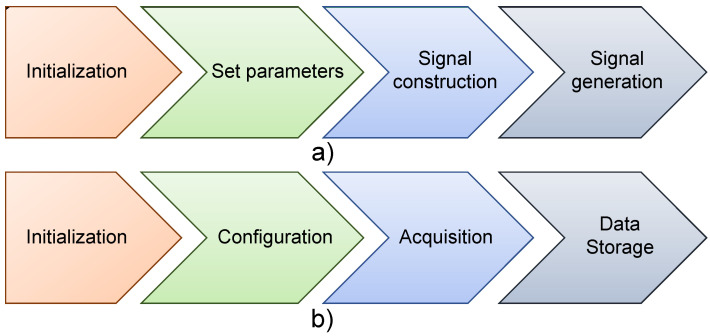
Scheme for: (**a**) Signal generator. (**b**) Data acquisition system.

The system was developed by using, as software, LabVIEW^™^. It considers two general parts, signal generator and data acquisition. In the first, described in Figure 4a, a tone burst signal is defined to be applied to the PZTs by using the hardware described in the paper. To achieve this, the procedure detailed below is followed:

- Initialization: Search and identification of device (arbitrary wave generator).
- Adjust parameters: The characteristics of the signal such as frequency and amplitude are fixed.
- Signal construction: The signal is constructed using trigonometric functions, normal distribution functions, and direct digital synthesis (DDS).
- Signal generation: The signal is generated and produced through the output channel of the generator.

In the second part, data acquisition and data storage in memory are carried out. The procedure is shown in Figure 4b and detailed below:

- Initialization: As in the first part, the search and identification of the device are carried out.
- Configuration: Data acquisition parameters, such as sampling frequency, horizontal scale, vertical scale, among others, are set.
- Acquisition: Data entering each of the channels of the data acquisition system are read.
- Storage: Data are stored in memory for processing.

**Figure 5:**
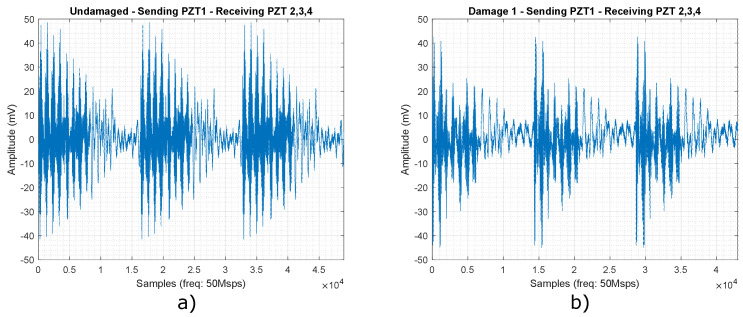
Received signals for actuation Phase 1 when: (**a**) there is no damage in plate and (**b**) Damage 1 is performed on the structure.

Figure 5 shows the signal collected for actuation phase 1, that is, when PZT1 is an actuator and PZT 2, 3 and 4 are sensors, for an undamaged plate (a), and the signal is collected when damage 1 is performed on the structure. Each response signal that is displayed in Figure 5 corresponds to PZT 2, 3 and 4. These signals are collected individually from each PZT and then concatenated through an organization and filtering process, where high-frequency noise and impulsive noise from activities outside the experiment are removed.

Figure 5a,b show a series of tone burst signal reflections that are attenuated over time. It is not always possible to detect damages in structures observing graphs like these; for this reason, this work focuses on the classification of damage from pattern recognition, where the changes that occur in the structure are detected by machine learning algorithms for any of the action phases.

## 3. Theoretical Background

The collected data need to go through a series of statistical pre-processing methods before being analyzed by classification algorithms. This data treatment involves the use of normalization techniques, which are responsible for scaling the data, establishing a unit amplitude, helping to eliminate noise, and minimizing the non-linearity associated with the sensors or the material of the structure under study.

After carrying out the normalization of the data, the principal component analysis (PCA) technique is applied to reduce the size of the data, which contributes to reducing the computational cost, and feature extraction (that is, the subtraction of the most relevant information in dataset). The information resulting from this process will constitute the input data to the classification algorithms.

### 3.1. Principal Component Analysis (PCA)

Normalization techniques help to establish a unit variance in the data, as far as possible, serve as filters, and eliminate redundancy. Therefore, applying normalization techniques improves the computational efficiency of the classification algorithms, however, a drawback persists, and there is no guide to determine which technique is indicated. For this reason, different attempts must be made with each one. A list of the different normalization techniques studied [55], with a brief description, and the respective formula, is given below.

#### 3.1.1. Autoscaling

Autoscaling allows one to adjust the mean of the data to the origin and the variance of each one by 1. It is often used in cases involving signals with a different magnitude scale [56].

(1) $$A_{ij}=\frac{A_{ij}-mean\left(A_{j}\right)}{std(A_{j})}$$

#### 3.1.2. Group Scaling

Group scaling considers the changes or differences between the different sensors, and does not process them independently [56].

(2) $$A_{ij}=\frac{A_{j}-mean\left(A_{j}\right)}{std(A_{j})}$$

#### 3.1.3. Relative Scale 1

Relative scale 1 performs a global compression of all values by setting a maximum value of 1 [56].

(3) $$A_{ij}=\frac{A_{ij}}{max(A)}$$

#### 3.1.4. Relative Scale 4

Relative scale 4 compresses the data according to the norm of the matrix containing all *n* samples for sensor *j* [56].

(4) $$A_{ij}=\frac{A_{ij}}{\left|\right|A_{i}\left|\right|}$$

#### 3.1.5. Range Scale 1

Range scale 1 adjusts the amplitude limits of the data between 0 and 1 [56].

(5) $$A_{ij}=\frac{A_{ij}-min\left(A_{j}\right)}{max\left(A_{j}\right)-min\left(A_{j}\right)}$$

#### 3.1.6. Range Scale 2

Range scale 2 adjusts the amplitude limits of the data between −1 and 1 [56].

(6) $$A_{ij}=\left[2\left(\frac{A_{ij}-min\left(A_{j}\right)}{max\left(A_{j}\right)-min\left(A_{j}\right)}\right)\right]-1$$

#### 3.1.7. Standard Normal Variate Transform (SNVT)

SNVT reduces data spread within each class [56].

(7) $$A_{ij}=A_{ij}=\frac{A_{ij}-mean\left(A\right)}{std(A)}$$

For all the above cases: *A* is the characteristics matrix for *n* samples of *p* sensors and $A_{ij}$ is the matrix of the *i*-th sample of the *j*-th sensor [56].

Principal component analysis (PCA) is a technique used in the elaboration of predictive models. One of the main advantages of its use is the reduction of the dimensions of the processed data by eliminating redundancies, but maintaining the variance of the data as much as possible. This is achieved by transforming the principal components into a new set of variables. These components are, at the beginning, uncorrelated and organized in such a way that the first accumulates the highest variance of all the original variables [57]. To elaborate a model in PCA, it is necessary to organize the data in a matrix X of size n×m (*m* sensors and *n* tests) [3]. After using the normalization techniques described above, the covariance matrix is calculated from the following equation:

(8) $$C_{x}=\frac{1}{n-1}X^{T}X$$

This covariance matrix $C_{x}$ has a size of m×m and measures the degree of linear relationship between the data and its variables. The values and eigenvectors of the matrix $C_{x}$ define the subspaces in PCA as follows:

(9) $$C_{x}\tilde{P}=\tilde{P}A$$

where the eigenvectors of $C_{x}$ are the columns of $\tilde{P}$ and the eigenvalues are the diagonal terms of A. It is necessary to clarify that the terms outside the diagonal are zero [57]. The columns of the $\tilde{P}$ matrix are arranged in descending order based on their eigenvalues, and are called principal components. The most important patterns within the dataset are represented by vectors and eigenvalues. The structure is modeled by constructing the transformation matrix, where a number of principal components, less than the number of trials (r<n), is selected. Matrix P is a reduced and better organized version of $\tilde{P}$; it is called the PCA model [57], and represents the projection of the original data on the address of the principal components P:

(10) T=XP

However, it is not possible to use the reduced matrix P with the matrix T to fully recover the matrix X.

### 3.2. Damage Detection Indices

By analyzing a structure that has been used for a specific application for which it was designed, it is possible to project a new model of this structure (when it has been subjected to continuous use) on the base model (without damage) and detect whether or not there have really been significant changes in its structural state or properties. However, sometimes these projections are not enough, and therefore, the use of statistical measurements that can be considered as damage indices is required. These indices are the *Q* or SPE (square prediction error) index, and Hotelling’s T${}^{2}$ index [58].

#### 3.2.1. Hotelling T${}^{2}$ Statistic

Hotelling’s T${}^{2}$ statistic is a derivation of the *t* statistic for hypothesis testing on multivariate data. The T${}^{2}$ statistic for each sample or experiment in the dataset is defined as follows:

(11) $$T_{i}^{2}=\sum_{j=1}^{r}\frac{t_{sij}^{2}}{\lambda_{j}}=t_{si}A^{-1}t_{si}^{T}=x_{i}PA^{-1}P^{T}x_{i}^{T}$$

where $x_{i}$ is the vector that represents the measurements of each experiment for each sensor [58] and $t_{si}$ is the projection of the experiment $x_{i}$. They are related as $t_{si}=x_{i}P$ [3,58].

#### 3.2.2. Q Statistic

The statistic or Q index expresses changes that cannot be explained by the PCA model as the difference between the sample and its projection in the model. The Q index for each sample or experiment of the vector $x_{i}$ is defined as follows:

(12) $$Q_{i}={\tilde{x}}_{i}{\tilde{x}}_{i}^{T}=x_{i}\left(I-PP^{T}\right)x_{i}^{T}$$

where ${\tilde{x}}_{i}$ is the projection on the residual subspace. As the magnitude of Q is very small compared with the index T${}^{2}$, Q turns out to be more sensitive, thus allowing us to observe any changes that occur in the system [58].

### 3.3. Damage Classification

Due to the growing needs that arise in structural health monitoring regarding classification methodologies that involve machine learning algorithms, the study and design of neural networks persist, with accurate and reliable results with increasingly better computational efficiency [37]. The use of more elaborated machine learning algorithms avoids the need for sophisticated computers that are not available to any laboratory or researcher. Some classification algorithms that address the use of neural networks based on self-organizing maps are explored below, including both supervised learning networks (SKN and XYF) and semi-supervised learning networks (CPANN).

#### 3.3.1. Self-Organizing Maps (SOM)

Self-organizing maps (SOM) represent a machine learning technique that uses a neural network that can be supervised, semi-supervised, or unsupervised. It is a type of technique widely used with many applications, and allows one to carry out data grouping and visualization tasks. To carry out their classification process and find similarities between data, self-organizing maps are based on metrics such as the Euclidean distance, which is employed to construct groupings of data that share similar properties [38,39,59]. Many models have been developed from the first descriptions made by Teuvo Kohonen in 1982; this article briefly describes the CPANN, SKN, and XYF networks.

#### 3.3.2. Counterpropagation Artificial Neural Network (CPANN)

**Figure 6:**
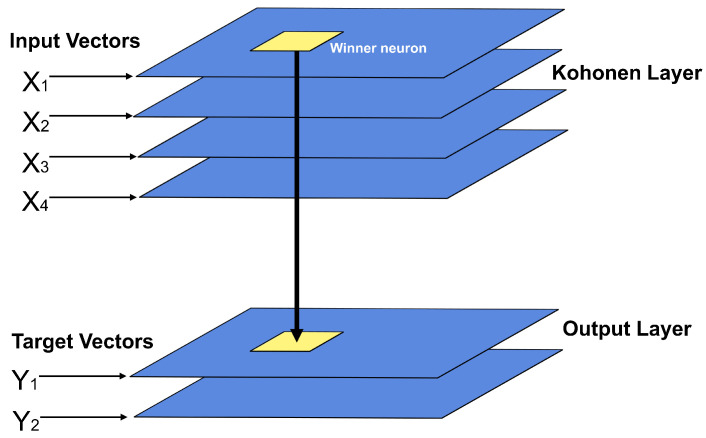
Counterpropagation Artificial Neural Network structure.

CPANN is a semi-supervised neural network that combines the self-organizing mapping technique and the Grossberg neural network. The built model contains one layer of each type of network mentioned. CPANN has generalization capabilities, which would allow dealing with input vectors that are incomplete or partially incorrect [41]. Figure 6 shows the structure of this type of neural network.

This neural network comprises an unsupervised network (SOM) and a supervised network (Grossberg). The layer corresponding to the Grossberg network is trained to converge to the desired output values (T), while the layer corresponding to SOM is trained to converge to average output values [40,41].

#### 3.3.3. Supervised Kohonen Network (SKN)

**Figure 7:**
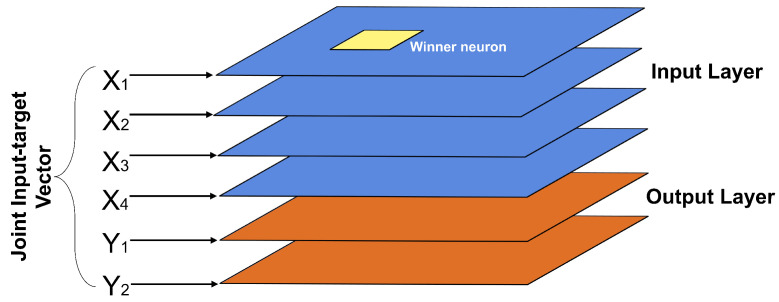
Supervised Kohonen network structure.

This is a supervised learning network, where an input map ($X_{map}$) and an output map ($Y_{map}$) join to form a combined input and output map ($XY_{map}$). Each input set X is linked to its respective output Y to serve as an input parameter to $XY_{map}$ [42]. Figure 7 shows the structure of this type of neural network.

#### 3.3.4. X–Y Fused Kohonen (XYF)

This algorithm explores the similarities of both the input map ($X_{map}$) and the output map ($Y_{map}$) in a simpler way than SKN. The winning neuron is determined using a similarity measure based on a weighted combination of the similarities between an object X and all units on the map $X_{map}$, and the similarities between the output objects Y and the units in the map $Y_{map}$. The fused similarity measure $S_{Fused}\left(i,k\right)$ can be expressed as:

(13) $$S_{Fused}\left(i,k\right)=\alpha \left(t\right)S\left(X_{i},X_{map-k}\right)+\left(1-\alpha \left(t\right)\right)S\left(Y_{i},Y_{map}\right)$$

**Figure 8:**
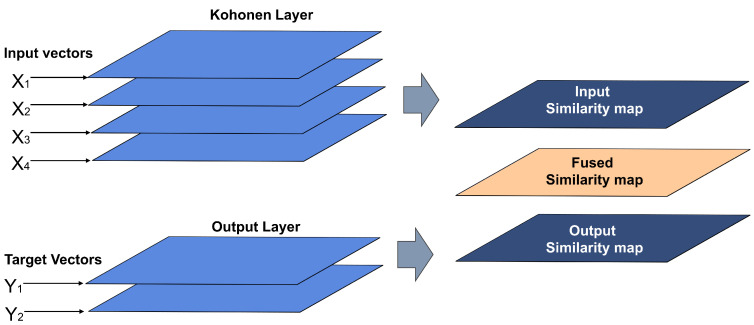
X–Y fused Kohonen network structure.

The minimum value in $S_{Fused}\left(i,k\right)$ defines the winning unit. The parameter α(t) deals with the relative weighting between similarities of the maps $X_{map}$ and $Y_{map}$. The *t* argument in α(t) is the number of iterations for training [42,60]. Figure 8 shows the structure of this type of neural network.

## 4. Proposed Methodology

The elements that are part of the methodology implemented in this work have been described in the previous sections. This methodology is composed of three main steps; 1. collection and storage of signals captured by the network of piezoelectric sensors; 2. statistical processing; and 3. damage classification.

### 4.1. Damage Classification Methodology

The damage classification methodology was developed to carry out the treatment of the data previously obtained with the data acquisition system (DAQ). The built methodology has the ability to perform the following tasks:

- Filtering, organization of the collected data, and construction of the data set. Since the DAQ control program only provided data in a .csv file, it is necessary to apply a filter that attenuates the noise (high-frequency noise and impulsive noise) and creates the matrices that will later be processed and analyzed. The filter bandwidth is defined to reject all the signals that are outside the spectrum of the tone burst signal shown in Figure 3.
- Normalization of the data set. Data scaling is carried out, eliminating the differences that could exist in the response generated by the different piezoelectric transducers. Although, in theory, they all have the same manufacturing characteristics, in practice, they turn out to be slightly different products of mass production.
- Reduction of the data set and feature extraction. Redundancy in the data is eliminated and statistical indices containing information on the structural changes of the object of study are extracted. This process is carried out using multivariate analysis techniques such as PCA (principal component analysis), which is explained in Section 3. After applying this technique, scores matrix, T${}^{2}$ and Q arrays are obtained; these elements are the features necessary to construct the input vectors for the neural networks.
- Damage classification by using supervised and semi-supervised self-organizing maps. This task is carried out using two supervised neural networks, as in the case of supervised Kohonen (SKN) and X–Y fused Kohonen (XYF), and only one semi-supervised algorithm, as in the case of counterpropagation artificial neural network (CPANN). At this stage, input vectors are created using PCA, and target vectors are used to train the previously mentioned neural networks.

**Figure 9:**
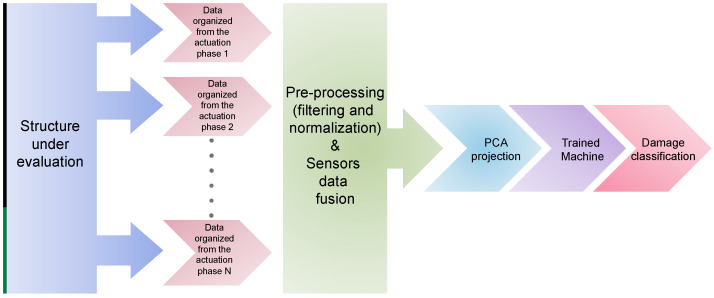
Classification methodology scheme.

Figure 9 shows the general scheme of the developed classification methodology.

### 4.2. General Scheme for Damage Classification

The classification methodology, as explained before, contemplates a pre-processing stage for data treatment, before implementing the final stage, where the data are analyzed using machine learning algorithms. Therefore, the procedure proposed to evaluate the structural monitoring system is as follows:

1. Piezoelectric transducer excitation.
2. Data collection.
3. Filtering and data organization.
4. Damaged and undamaged plate reference model construction.
5. Self-organizing maps training.
6. Results display.

**Figure 10:**
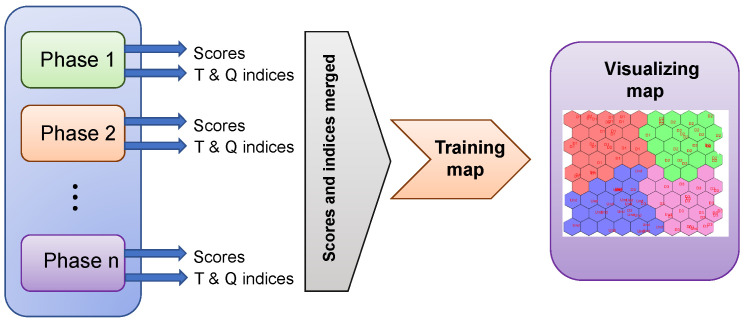
Classification methodology using self-organizing maps.

In order to build a single data set and a unified pattern of the data collected from the plates in the study, the scores and damage indices corresponding to each actuation phase were merged as shown in Figure 10.

Figure 10 shows the inputs to the classifier. As is shown, scores and T${}^{2}$ and Q indices (features) from each actuation phase are used as input for the classification algorithm. Since four actuation phases are implemented (one for each PZT, as shown below), then four scores matrices are obtained; four arrays corresponding to the Q index and four arrays corresponding to the T${}^{2}$ index. By merging the scores matrix with the T${}^{2}$ and Q indices, the input vectors are built, with which the CPANN, SKN, and XYF neural networks are trained. The target vector consists of an array that contains the labels, which are necessary in supervised learning, since they represent the answer that the machine learning model should predict. Its construction is often manual, and the structure of the dataset must be known for this, that is, of *n* samples that make up a dataset; a certain amount of these samples correspond to a structure without damage, and the same applies to the other structural states. To elaborate the input vectors, it is not necessary to include all the features obtained during the pre-processing stage; however, after many tests, it was determined that the more features that are implemented, the more robust the created model will be, and therefore better results will be obtained. It is important to clarify that the size of the scores matrix is proportional to the number of principal components selected; the criteria for selecting this number is detailed in Section 5.

**Figure 11:**
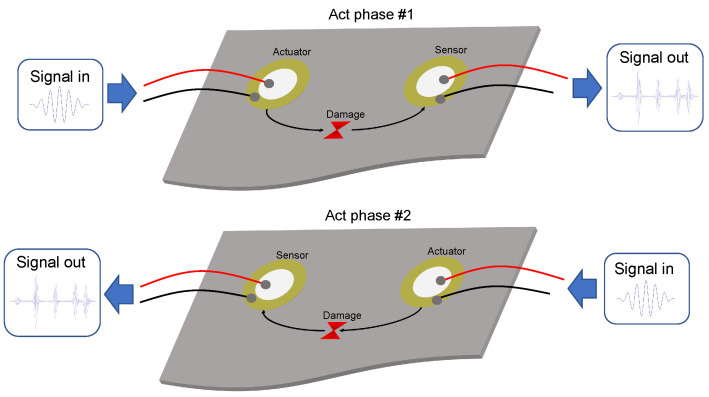
Structural monitoring procedure implementing different actuation phases.

The experiment is carried out through actuation phases, as explained in Section 2. It consists of rotating the actuator function among the different piezoelectrics that comprise the sensor network. Figure 11 shows this procedure in a very simplified way, and any damage in the structure should be detectable, even if the position of these is outside the area covered by the sensors, as shown in the first experiment in Section 5. The number of phases will depend on the number of sensors with which the plate or structure under study has been instrumented.

A large number of maps were trained in order to determine the best configuration, that is, parameters such as: adequate size of the map, normalization, initialization algorithms, classification, among others, allow well-defined data clustering in the maps to form, where each grouping of data would correspond to a different structural state.

The first step consisted of determining the size of the map, which depends mainly on the size of the dataset, that is, more data; the map should be a larger size in order to avoid overlaps between the different groupings of data that determine the classification algorithms [3,61]. All SOM neural networks were trained from 100 to 1000 learning epochs, and the dimensions of the networks varied from 4 × 4 to 12 × 12 neurons. The best models correspond to a dimension of 10 × 10 neurons. Based on the above, it was determined that the best pre-processing and configuration for the study cases treated in this article are the following:

- Pre-processing: Autoscaling.
- Initialization algorithm: Random.
- Training algorithm: Sequential.
- Neighborhood function: Gaussian.

This configuration refers to normalization techniques applied in the pre-processing and adjustable parameters of the machine learning algorithms. These machine learning algorithms were developed by Milano Chemometrics and QSAR Research Group of the Milano-Bicocca University in Milan, Italy [62,63,64]. For the three cases studied, the configuration shown turned out to be the one with the best results. The damages were simulated as masses adhered to the plate, in order to avoid permanent damages that would prevent their use in future experiments, however, as shown in Section 5, real damages were studied in a composite material plate (CFRP).

## 5. Results and Discussion

In order to validate the methodology proposed in Section 4, the results for damage classification in two aluminum plates and one composite material plate are presented.

### 5.1. Specimen 1: Aluminum Plate 200 × 200 mm

**Figure 12:**
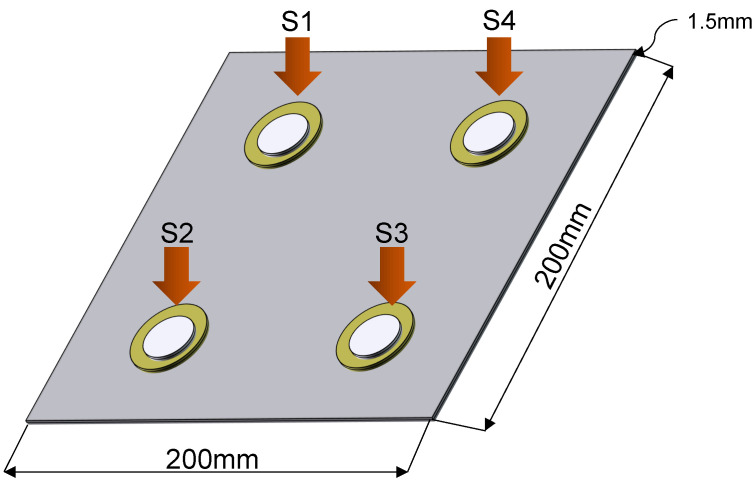
Graphic representation of the plate.

The first experiments were carried out with an aluminum plate of dimensions 200 × 200 mm and a 1.5-mm thickness, instrumented with four piezoelectric transducers made of copper and ceramic with a diameter of 27 mm, as shown in the representation graph of Figure 12. To carry out this experiment, it was necessary to develop a support for the plate that isolates it from external vibrations; additionally, this support maintains the plate in vertical position. The intention of keeping the plate in a vertical position lies mainly in the fact of facilitating its handling during the experiment; no significant changes would be expected if its inclination varies.

**Figure 13:**
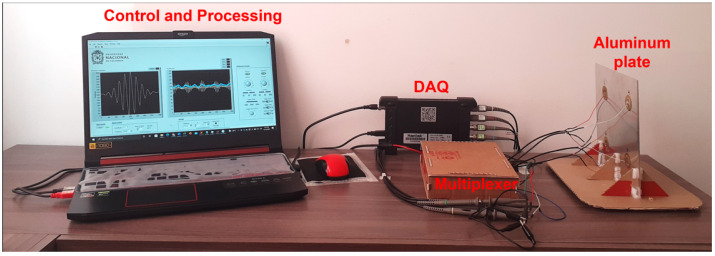
Experimental setup.

Data collection was carried out with a data acquisition software, developed with a total of four actuation phases. For each phase, 20 experiments were carried out; that is, as the number of times the sequence was carried out: send signal, receive response, and store data. The excitation signal was tone burst type, as illustrated in Figure 3, with a frequency of 500 kHz and amplitude $7V_{pp}$. To determine the signal frequency, it was necessary to perform a frequency sweep in such a way that the resonant peaks in the plate could be determined. The experimental setup is shown in Figure 13.

Two experiments were carried out with this plate. The first one contains two damages, and in the second one, a third damage is added in order to evaluate if the classification methodology is capable of classifying new damages that may appear, and to observe how the map is affected. As mentioned before, these damages were simulated as masses adhered to the structure, which consist of two neodymium magnets and a coin. Each of these objects represents damage, and their purpose is to cause disturbances in the signal collected. After being analyzed by the classification methodology, they indicate the presence of plate damage.

**Figure 14:**
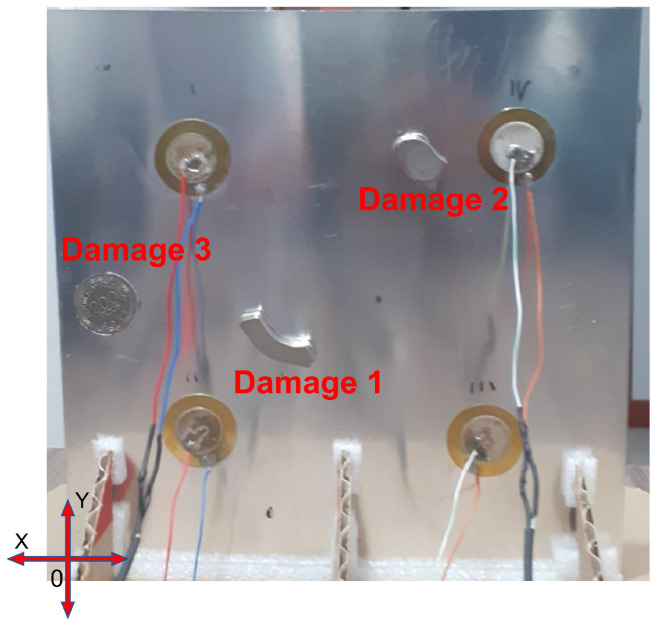
Damages position in the aluminium plate.

**Figure 15:**
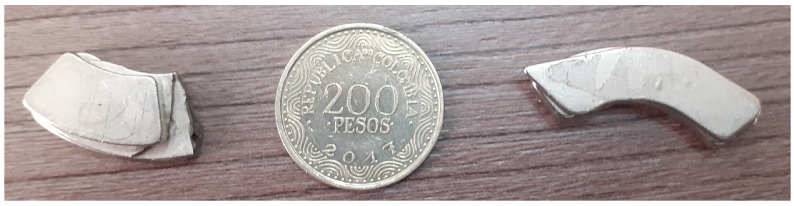
Elements used to simulate damages.

Figure 14 describes the position of the masses (damage) on the plate, in which a reference coordinate axis is established to allow the position to be indicated using distance measurements, as displayed in Table 1. As shown in Figure 14, the mass that represents each damage adhered to the structure. In the case of magnets, it is necessary to locate one on each side of the plate to hold it, since aluminum is not a ferromagnetic material. In the case of the coin, it can be attached using glue or magnets as well, however, for this experiment, it is attached with glue. It should be noted that the three damages are not studied simultaneously, but sequentially; that is, the mass corresponding to damage 1 is placed, and the plate is inspected with the piezoelectric network, then the mass corresponding to damage 2 is placed and the plate is inspected again, and this process is repeated according to the amount of damage that is to be simulated. The same procedure applies if real damages are studied. Figure 15 shows, in more detail, the elements used for the damage simulation.

Once the dataset is obtained, it was processed by pre-processing, and classification algorithms previously explained. Each dataset has a number of rows and columns, the rows represent the number of samples collected from the plate for each structural state, it is very important that the number of samples allows extracting sufficient information for the classification of a new structural state without a very extensive dataset. In these experiments, 20 samples were allowed to obtain satisfactory results.

**Figure 16:**
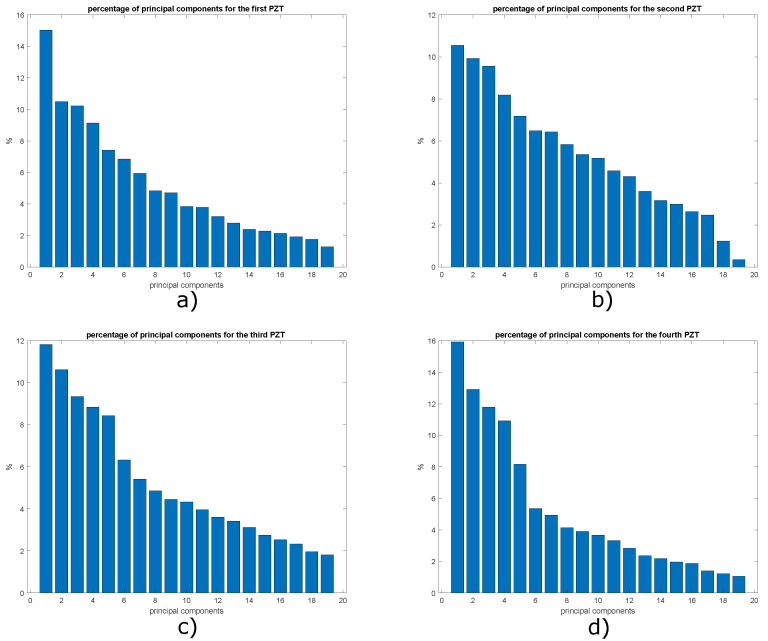
Variance distribution in: (**a**) phase 1; (**b**) phase 2; (**c**) phase 3 and (**d**) phase 4.

A necessary task during the development of this work was to determine what is the suitable number of principal components for the construction of the representative models of the plate with and without damage. To achieve the above, graphs were developed to visualize the percentage of cumulative variance by the 38 principal components. The graphs of the distribution of variance percentages between the principal components are shown in Figure 16 for the four actuation phases.

In order to determine what is the necessary percentage of cumulative variance to build an adequate pattern that later allows obtaining satisfactory classification results, models with 2, 4, 6, and 8 principal components were created based on the graphs in Figure 16. With eight principal components, a cumulative percentage of around 50% was achieved. The importance of this value is due to the fact that it expresses the amount of information that each principal component is capable of retaining. More principal components could be included, and a higher percentage and therefore better classification results could be achieved, however, this would mean creating bigger feature vectors which would increase the processing time using machine learning algorithms. The results obtained for the plate with two and three damages are presented below.

#### 5.1.1. Case Study No. 1: Aluminum Plate with Two Damages

**Figure 17:**
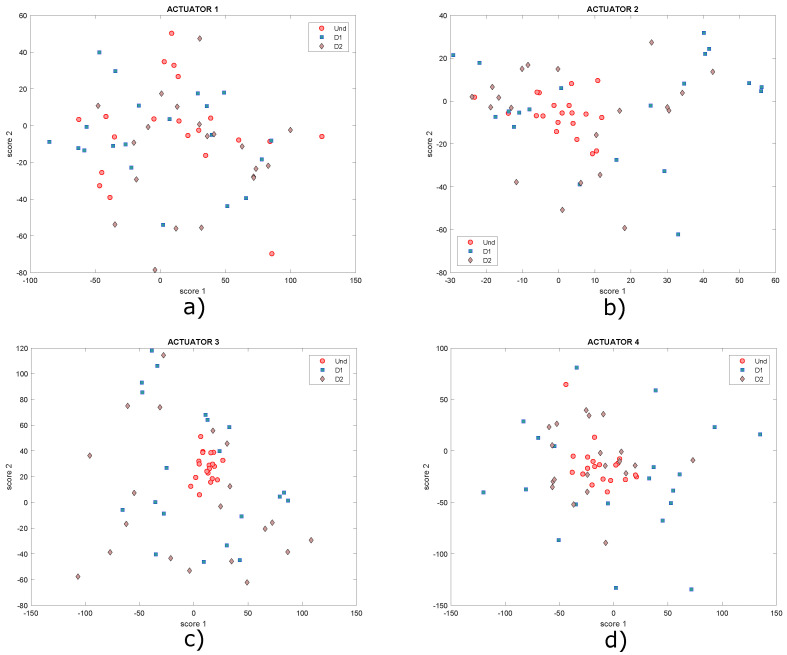
Graphs of score 1 vs. score 2 for actuation phases: (**a**) 1; (**b**) 2; (**c**) 3 and (**d**) 4.

Two neodymium magnets (D1, D2) shown in Figure 15 are the elements that simulate damages on the plate in this case. The aim of this kind of damage is to change the mass of the structure and affect the propagation of the waves produced by the piezoelectric transducers. The location of each one is indicated in Table 1. After applying the autoscaling normalization technique (with which the best result was obtained), the principal component analysis (PCA) technique was applied, as explained in Section 3 and Section 4. From this technique, the models of the undamaged plate and the damaged plate are obtained in three matrices called Scores and indices T${}^{2}$ and Q. By projecting the model of the undamaged plate on the model of the damaged plate, a new model is obtained with the same three matrices already mentioned. The scores matrix contains the principal components and allows constructing a series of scatter plots that allow visualizing the structural state of the plate. In the two-dimensional form, the first two principal components (scores 1 vs. scores 2) are plotted for each actuation phase. Since the structural state is known in each experiment carried out, it is then possible to label the data with the purpose of improving the representation of the results by identifying each group with a different color. For the plate under study, the score graphs in Figure 17 were obtained for different actuation phases. In these graphs different structural states are represented by points of different shapes and colors to make the distinction.

**Figure 18:**
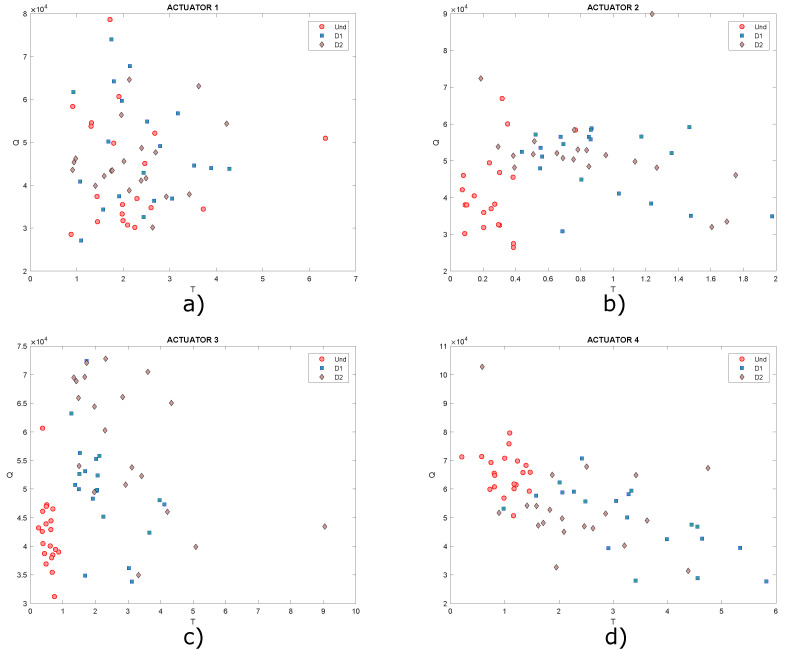
Graphs of T${}^{2}$ vs. Q for actuation phases: (**a**) 1; (**b**) 2; (**c**) 3 and (**d**) 4.

As shown in the graphs, it is not possible to determine a specific pattern that allows the different structural states to be identified since the data are mixed together, ideally it would be expected that for each structural state, a cluster of points would be created. Under these circumstances, it is necessary to implement classification algorithms in order to improve the grouping of data. Another type of scatter plot is T${}^{2}$ vs. Q, which are damage indices that can also be plotted in order to determine patterns in the data, as they contain information on any changes that the structure or plate under study may have experienced. Below, in Figure 18, the graphs obtained from T${}^{2}$ vs. Q for the different actuation phases are presented.

In this Figure, data are very dispersed, and in some cases, it is mixed, as already mentioned, ideally it is expected that clusters of points will be created, where each cluster would represent a different structural state. Only data corresponding to undamaged state could be highlighted in Figure 18b,c. Based on the above, it is useful to implement self-organizing maps to achieve a better grouping of these data.

**Figure 19:**
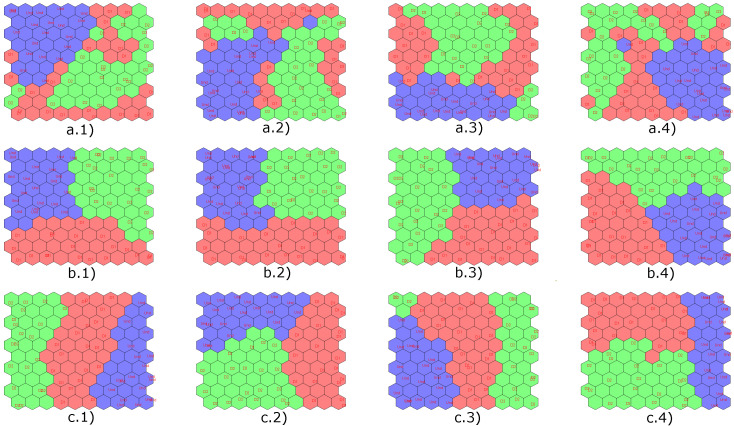
Results obtained by implementing the CPANN (**a**), SKN (**b**) and XYF (**c**) networks for: 2 principal components (group 1), 4 principal components (group 2), 6 principal components (group 3) and 8 principal components (group 4).

Next, in Figure 19, the results obtained with the implementation of the CPANN, SKN and XYF neural networks are presented, showing that the number of principal components does not affect the final result for the last two neural networks. In these figures, the structural states are represented by different colors: blue is for undamaged state, red is for damage 1, and green is for damage 2.

#### 5.1.2. Case Study No. 2: Aluminum Plate with Three Damages

**Figure 20:**
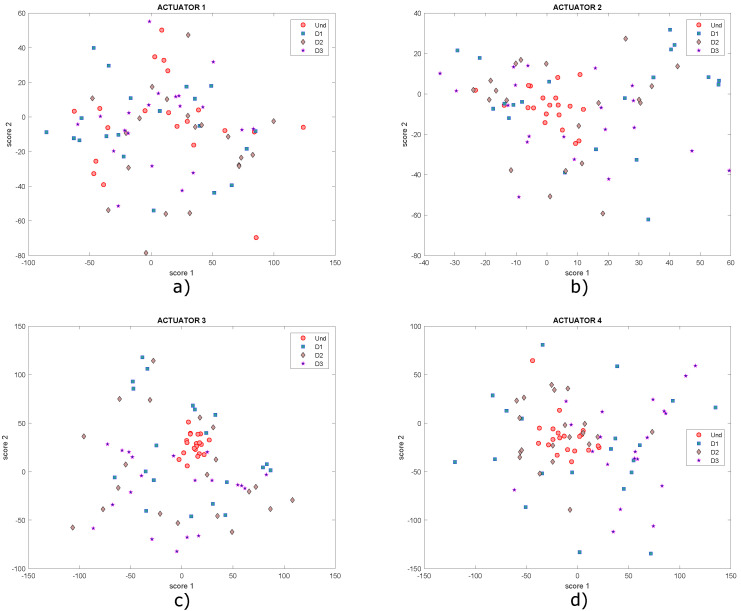
Graphs of score 1 vs. score 2 for actuation phases: (**a**) 1; (**b**) 2; (**c**) 3 and (**d**) 4.

The data collection procedure is carried out again. Now, a third damage (D3) is produced with a mass of different characteristics such as a coin, attached to the structure and located as indicated in Table 1 and illustrated in Figure 14. The data on this damage were added to the dataset constructed from the experiments described in the previous subsection. After applying the normalization and PCA, the undamaged and damaged plate models were again extracted and the T${}^{2}$ and Q scores and indices were graphed. Figure 20 shows the graphs of scores 1 and 2 with a new damage (D3).

**Figure 21:**
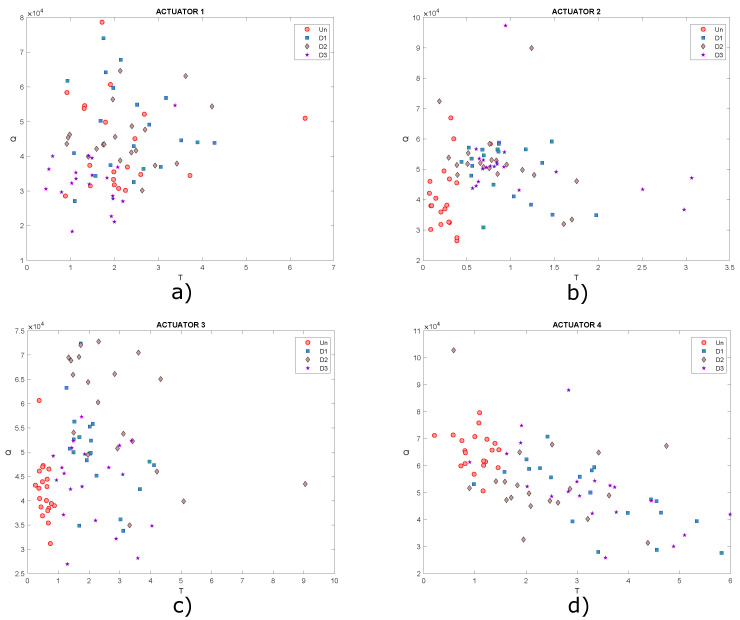
Graphs of T${}^{2}$ vs. Q for actuation phases: (**a**) 1; (**b**) 2; (**c**) 3 and (**d**) 4.

These graphs show that it is not possible to visualize well-defined groupings in the dataset. The same happens graphing T${}^{2}$ vs. Q, as shown in Figure 21, where a grouping of the data corresponding to damages 2 and 3 can hardly be identified in Figure 21b. However, in the rest of the graphs, data are very dispersed in addition to being mixed with each other. It is expected that clusters of points will be created, where each cluster would represent a different structural state, but this is not the case.

**Figure 22:**
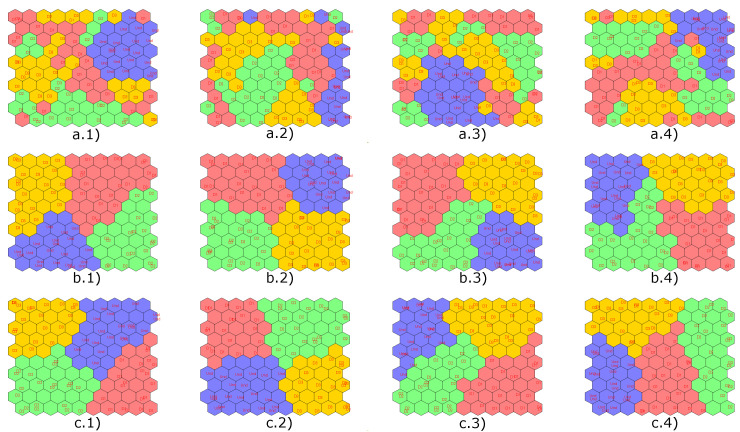
Results obtained by implementing the CPANN (**a**), SKN (**b**) and XYF (**c**) networks for: 2 principal components (group 1), 4 principal components (group 2), 6 principal components (group 3) and 8 principal components (group 4).

Results obtained by applying semi-supervised (CPANN) and supervised (SKN and XYF) algorithms are presented below. Figure 22 shows the results of the classification process produced by the aforementioned networks using a dataset with four scores (one per actuation phase), and T${}^{2}$ and Q indices. In Figure 22, the structural states are represented by different colors: blue is for undamaged state, red is for damage 1, green is for damage 2 and yellow is for damage 3.

As it is shown in Figure 22, the data group corresponding to the structural state damage 1 and damage 2 is not very well defined for CPANN network. From all the graphs with results of the classification process carried out by the SKN and XYF networks, it can be determined that the number of principal components is not a key factor in the effectiveness of the results. At least two principal components are enough so that they can form well-defined and separate datasets.

In order to evaluate the performance of the CPANN, SKN and XYF networks, a comparative analysis is carried out with other types of neural networks: K-nearest neighbors (kNN), classification trees (CART), partial least square discriminant analysis (PLSDA), discriminant analysis (DA), and backpropagation neural networks (BPNN). This comparison used the MATLAB Classification Toolbox developed by Milano Chemometrics and the QSAR Research Group of the Milano-Bicocca University in Milan, Italy [62,63,64]. This is the same team that developed the Self-Organizing Maps toolbox used in this study for damage classification. As shown in Table 2, XYF and SKN networks present the best performance with an overall accuracy of 73.75% and 72.5%, respectively. To obtain these results, a cross-validation was carried out by applying a method that both toolboxes had in common: venetian blinds. It is a type of k-fold where each test set is determined by selecting every *i*-th sample in the dataset, starting at samples numbered 1 through *i* [65]. For this cross-validation procedure, five folds were implemented, because a high number of folds, which means more computation time, however largely irrelevant.

### 5.2. Second Specimen: Aluminum Plate 400 × 400 mm

**Figure 23:**
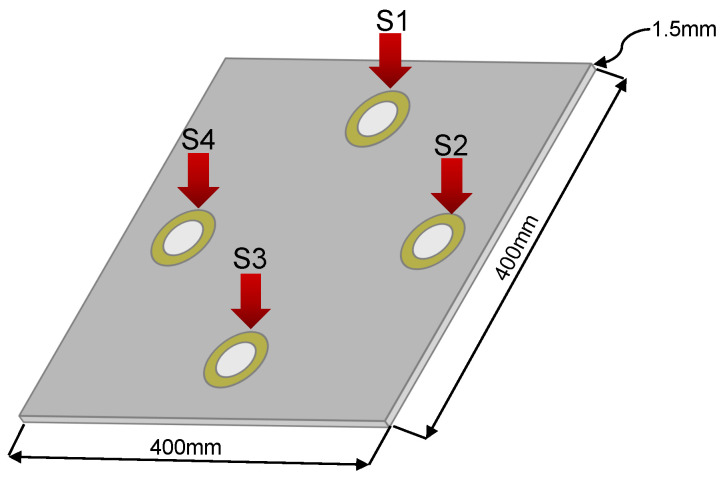
Graphic representation of the second specimen plate.

The second specimen studied in this paper was a 400 × 400 mm and a 1.5-mm thickness aluminum plate instrumented with four 35 mm diameter piezoelectrics, distributed in a different way from the first plate and its graphic representation is shown in Figure 23.

**Figure 24:**
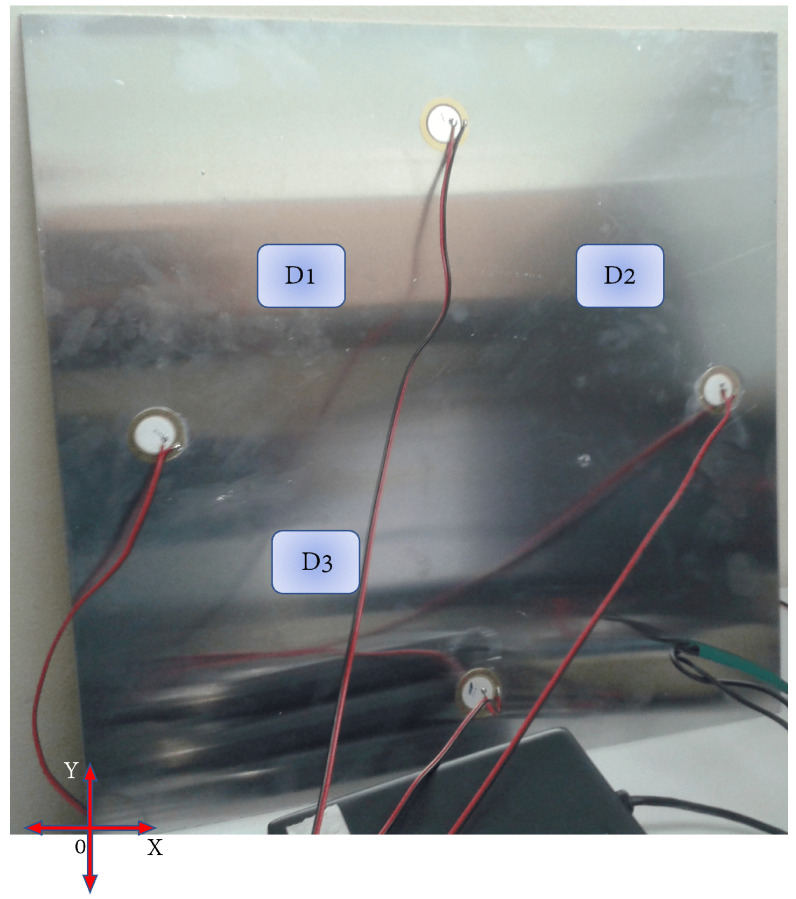
Damage position in the aluminium second specimen plate.

Damages were simulated as masses adhered to the structure whose location is indicated in Table 3 based on the coordinate axis shown in Figure 24, which also illustrates the location of damage on the board. With this plate, 25 experiments were carried out in each action phase for each structural state. The piezoelectrics were excited with a tone burst signal with the same characteristics, as in the first experiment presented.

**Figure 25:**
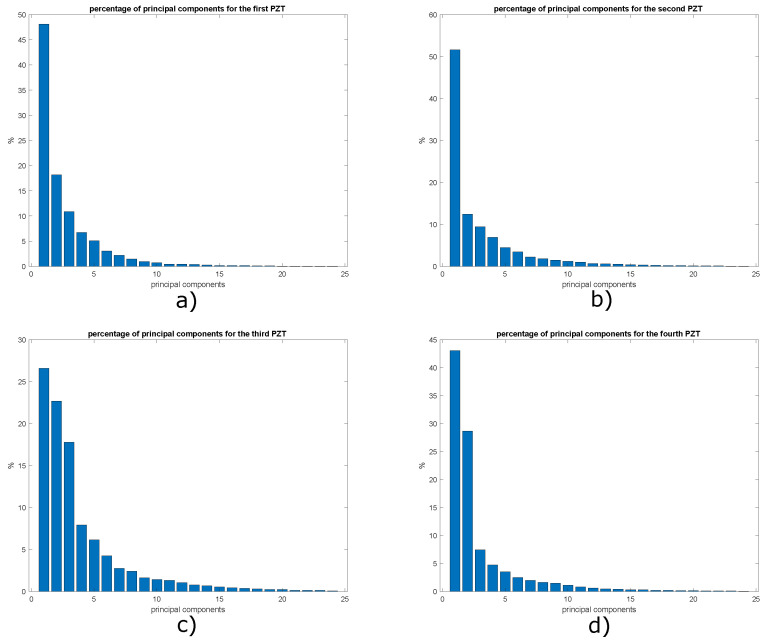
Variance distribution in: (**a**) phase 1; (**b**) phase 2; (**c**) phase 3 and (**d**) phase 4.

**Figure 26:**
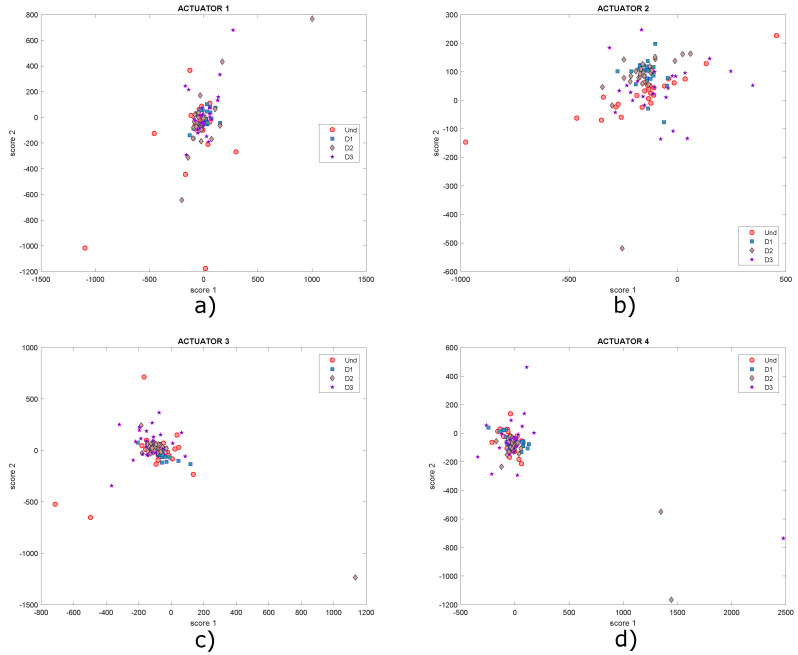
Graphs of score 1 vs. score 2 for actuation phases: (**a**) 1; (**b**) 2; (**c**) 3 and (**d**) 4.

**Figure 27:**
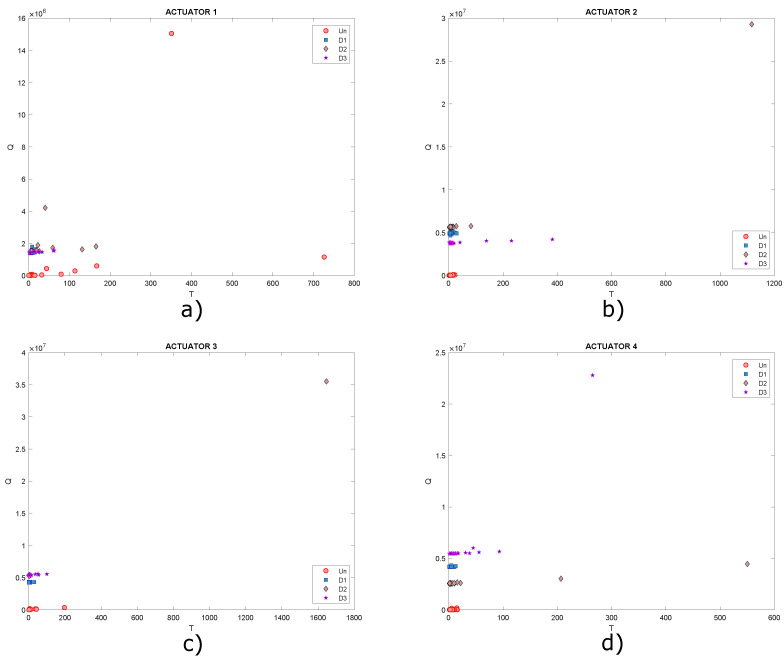
Graphs of T${}^{2}$ vs. Q for actuation phases: (**a**) 1; (**b**) 2; (**c**) 3 and (**d**) 4.

As shown in Section 5.1, the percentage of cumulative variance presented in Figure 25 is evaluated to determine an appropriate maximum number of principal components. A total of eight principal components contain 90% of the accumulated variance, and the results obtained are compared with 2, 4 and 6 principal components. Figure 26 shows that the graphs of score 1 vs. score 2 and Figure 27 present the graph for damage indices T${}^{2}$ vs. Q, where data groups belonging to an specific structural state are relatively separated from each other. This is the result that would be expected to find in this type of graphs. However, this matter can be improved by the implementation of self-organizing maps as illustrated in Section 5.1.

**Figure 28:**
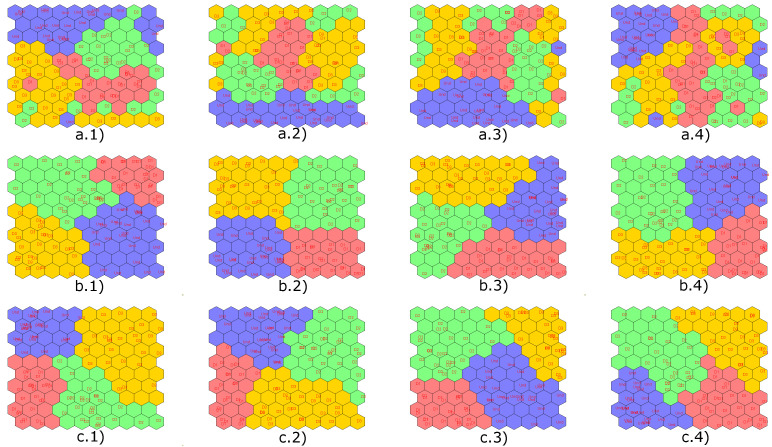
Results obtained by implementing the CPANN (**a**), SKN (**b**) and XYF (**c**) networks for: 2principal components (group 1), 4 principal components (group 2), 6 principal components (group 3) and 8 principal components (group 4).

Next, Figure 28 presents the results obtained with the implementation of the CPANN, SKN and XYF neural networks. In Figure 28, the structural states are represented by different colors: blue is for undamaged state, red is for damage 1, green is for damage 2, and yellow is for damage 3.

Once again, the CPANN network did not have satisfactory classification results compared to SKN and XYF networks. With these results, it is verified that the number of principal components has no significant influence on the classification result for the SKN and XYF networks, at least two principal components are required to achieve satisfactory results.

### 5.3. Third Specimen: Composite Material (CFRP) Plate

**Figure 29:**
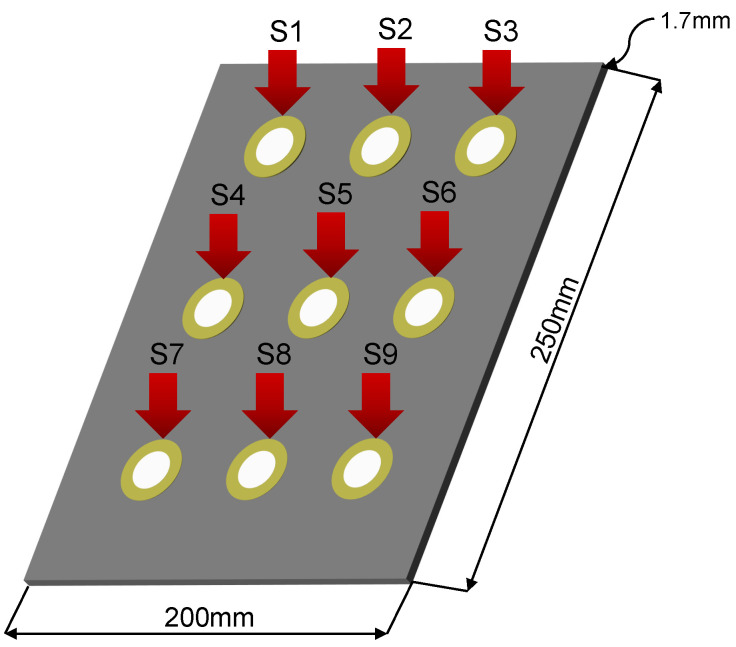
Graphic representation of the third specimen plate.

The third specimen studied is a composite material plate (CFRP) with dimensions 250×200 mm and a 1.7-mm thickness, made with four layers and instrumented with nine piezoelectric elements of 15mm diameter distributed as shown in Figure 29.

**Figure 30:**
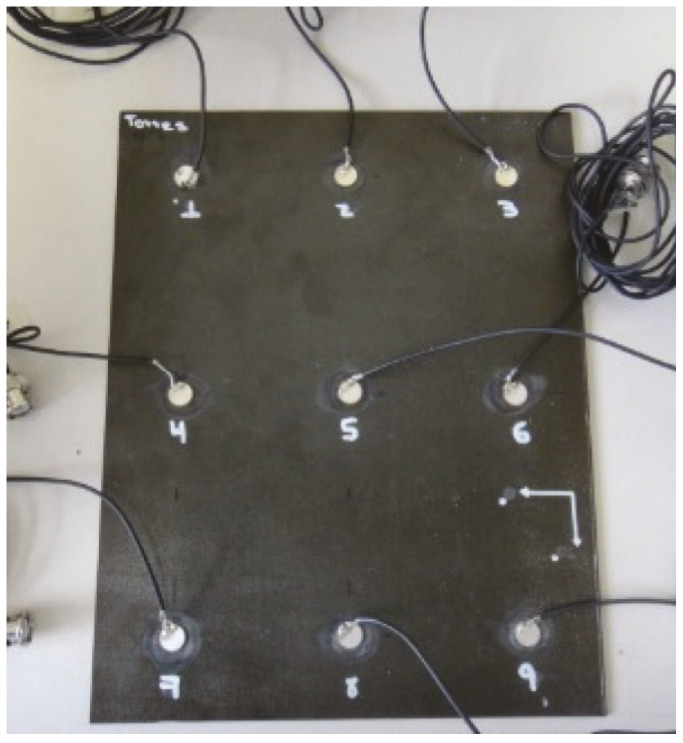
Third specimen, Carbon Fiber Reinforced Polymer plate.

The aim of this experiment is the validation of the methodology by applying real damages on the plate. Details about dimensions and distribution of the sensors can be found in Figure 29. Table 4 describes the number and kind of damages applied to the structure shown in Figure 30. For this plate, 20 experiments have been carried out in each action phase for each structural state as in the previous experiments. The excitation signal used is a tone burst type with amplitude of 12$V_{pp}$ and frequency of 30 kHz, which was determined from a frequency sweep.

**Figure 31:**
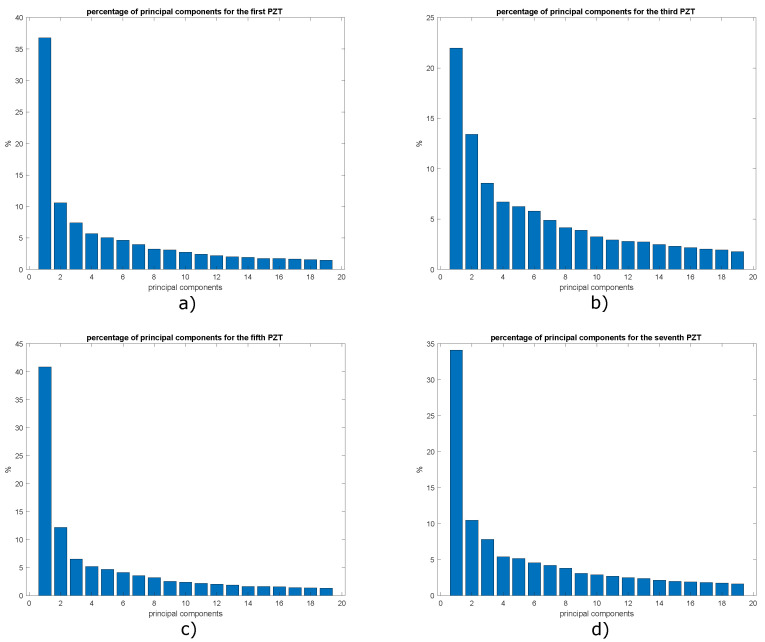
Variance distribution in: (**a**) phase 1; (**b**) phase 2; (**c**) phase 3 and (**d**) phase 4.

To determine the number of principal components necessary to build a model with enough information about the structural state of the plate, it is necessary to calculate the percentage of cumulative variance by each principal component, to achieve this, the graphs shown in Figure 31 have been constructed. It is important to clarify that only graphs corresponding to actuation phases 1, 3, 5 and 7 are shown, since they present the most relevant results.

A percentage of cumulative variance of approximately 77.5% was achieved with 8 principal components, as previous experiments this is the maximum value of principal components in order to avoid too large input vectors for the Neural Networks studied. However, the classification methodology for 2, 4 and 6 principal components is also evaluated.

**Figure 32:**
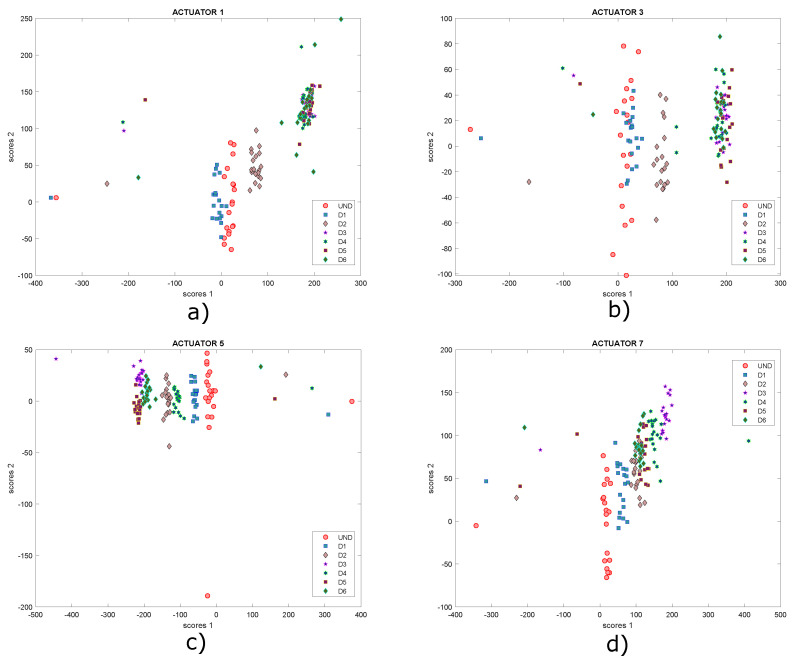
Graphs of scores 1 vs. scores 2 for actuation phases: (**a**) 1; (**b**) 3; (**c**) 5 and (**d**) 7.

**Figure 33:**
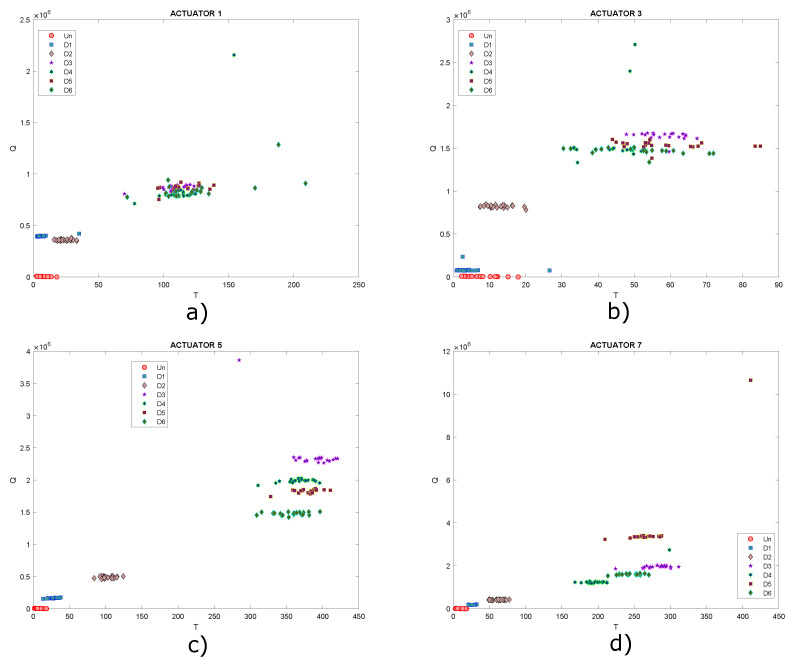
Graphs of T${}^{2}$ vs. Q for actuation phases: (**a**) 1; (**b**) 3; (**c**) 5 and (**d**) 7.

Figure 32 shows the graphs of scores 1 vs. scores 2 and Figure 33 presents the graph for damage indices T${}^{2}$ vs. Q, where data groups belonging to a specific structural state are relatively separated from each other. Although it is possible to see different groupings of data for each structural state (each one differentiated by points with a certain color and shape), this issue can be improved with the use of self-organizing maps as illustrated in previous experiments. As before, only results for actuation phases 1, 3, 5 and 7 are shown.

**Figure 34:**
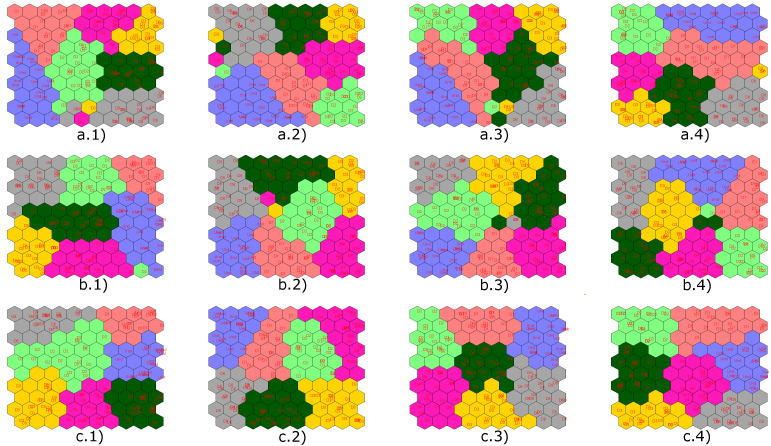
Results obtained by implementing the CPANN (**a**), SKN (**b**) and XYF (**c**) networks for: 2 principal components (group 1), 4 principal components (group 2), 6 principal components (group 3) and 8 principal components (group 4).

Next, Figure 34 presents the results obtained with the implementation of the CPANN, SKN, and XYF neural networks. In Figure 34, the structural states are represented by different colors: blue is for undamaged state, red is for damage 1, green is for damage 2, yellow is for damage 3, magenta is for damage 4, dark green is for damage 5 and gray is for damage 6. This results are obtained using features from all actuation phases.

Unlike the previous experiments, for this specimen, the CPANN network gave better results in terms of data grouping corresponding to different structural states.

## 6. Conclusions and Future Work

A structural monitoring system was proposed for damage classification in metal and composite material structures based on normalization techniques, multivariate analysis, such as PCA, and supervised self-organizing maps. The experiments were carried out on two aluminum plates with different characteristics and instrumented with four piezoelectric sensors each one, and one composite material (CFRP) plate instrumented with nine piezoelectric sensors. The classification methodology was tested with a small-scale experimental setup, where damages were simulated as masses adhered to the structure for aluminum plates, and real damages were assessed in composite material plate. This methodology is based on supervised artificial neural networks which have been little explored in classification tasks for SHM thus highlighting the relevance of this work.

The main conclusions of this work are:

- To classify a damage it is necessary firstly to detect the damage. In this case the scores and indices allowed to know the differences in the data from the healthy structure with the unhealthy structure or with abnormal conditions. These features from each actuation phase are used as input to the classifier to classify different conditions of the structure including the healthy state.
- Data preprocessing: normalization techniques are supposed to be a necessary step in classification tasks. Seven techniques presented in Section 3 were explored, however, autoscaling produced the best results. This can be attributed mainly to the ability of this technique to adjust signals from different piezoelectrics with different scale and magnitude.
- Dimensionality reduction: principal component analysis allowed feature extraction by reducing the size of the dataset used in 99.92%. The selection of the number of principal components was based on the percentages of accumulated variance by selecting a number of components that retained at least 50% of the variance, which resulted in a total of 8 principal components.
- Damage classification: Three artificial neural networks based on self-organizing maps were evaluated: two with supervised learning (SKN and XYF) and one with semi-supervised learning (CPANN). The performance of each one was compared with other state-of-the-art algorithms in SHM, such as, k-nearest neighbors (kNN), Classification trees (CART), partial least square discriminant analysis (PLSDA), discriminant analysis (DA) and backpropagation neural networks (BPNN). According to the cross-validation results, the SKN and XYF networks are the best option, since their accuracy percentages were the higher, 72.5% and 73.75%, respectively. However, the validation of the CPANN network must continue, since it turned out to have a lower performance (accuracy of 61.25%) than several classification algorithms with which it was compared. Additionally, classification results show that at least two principal components are required to show satisfactory results for SKN and XYF in first experiment. Thus, these neural networks require features that accumulate less than 50% of the cumulative variance.

Future work will involve three important aspects: (i) validation of the classification methodology when the plate is subjected to temperature variations; (ii) validation of the classification methodology with structures with different characteristics (composition, size, shapes, etc.) from those already studied; and (iii) evaluation of other multivariate analysis techniques such as hierarchical nonlinear principal component analysis (h-NLPCA), and independent component analysis (ICA), among others.

## Figures and Tables

**Table 1:** Damage description.

Damage Number	Position in X [mm]	Position in Y [mm]	Object
1	80	70	Neodymium magnet
2	130	150	Neodymium magnet
3	20	95	Coin

**Table 2:** Comparison of SOM Neural Networks with others.

Neural Netwok	Training		Cross-Validation	
	Accuracy %	Error %	Accuracy %	Error %
CPANN	92.5	7.5	61.25	38.75
SKN	100	0	72.5	27.5
XYF	100	0	73.75	26.25
kNN	60	40	65	35
CART	82	17	65	35
PLSDA	83	17	66	34
DA	99	1	56	44
BPNN	100	0	45	55

**Table 3:** Damage description in the second specimen aluminium plate.

Damage Number	Position in X [mm]	Position in Y [mm]	Object
1	100	250	Magnet
2	250	250	Magnet
3	100	100	Magnet

**Table 4:** Damage description in third specimen, composite material plate.

Damage Number	Damage Description
1	Delamination with dimensions 16×10 mm
2	Previous damage extended to 33×42 mm
3	Crack of 25 mm
4	Previous crack extended to 30 mm
5	Previous crack extended to 45 mm
6	Previous crack extended to 70 mm

## Data Availability

The data presented in this study are available from the corresponding author.

## Abbreviations

The following abbreviations are used in this manuscript:

BPNN	Backpropagation Neural Networks
CART	Classification Trees
CFRP	Carbon Fiber Reinforced Polymer
CPANN	Counterpropagation Artificial Neural Network
DA	Discriminant Analysis
DAQ	Data Acquisition System
kNN	k-Nearest Neighbours
PCA	Principal Component Analysis
PLSDA	Partial Least Squares Discriminant Analysis
PZT	Piezoelectric
SKN	Supervised Kohonen Network
SOM	Self-Organizing Map
XYF	X–Y Fused Kohonen

## References

[1] Rytter A. (1993). Vibrational Based Inspection of Civil Engineering Structures. Ph.D. Thesis.

[2] Farrar C., Worden K. (2012). Sensing and Data Acquisition. Structural Health Monitoring.

[3] Tibaduiza D. (2013). Design and Validation of a Structural Health Monitoring System for Aeronautical Structures. Ph.D. Thesis.

[4] Anaya M., Tibaduiza D.A., Torres-Arredondo M.A., Pozo F., Ruiz M., Mujica L.E., Rodellar J., Fritzen C.P. (2014). Data-driven methodology to detect and classify structural changes under temperature variations. Smart Mater. Struct..

[5] Yuan F., Yuan F.G. (2016). 1—Integrated vehicle health management in aerospace structures. Structural Health Monitoring (SHM) in Aerospace Structures.

[6] Malekloo A., Ozer E., AlHamaydeh M., Girolami M. (2021). Machine learning and structural health monitoring overview with emerging technology and high-dimensional data source highlights. Struct. Health Monit..

[7] (2021). Application of SHM Systems. https://www.acellent.com/applications-2.

[8] Comisu C.C., Taranu N., Boaca G., Scutaru M.C. (2017). Structural health monitoring system of bridges. Procedia Eng..

[9] Sousa H., Félix C., Bento J., Figueiras J. (2011). Design and implementation of a monitoring system applied to a long-span prestressed concrete bridge. Struct. Concr..

[10] Wang S., Wu W., Shen Y., Liu Y., Jiang S. (2020). Influence of the pzt sensor array configuration on lamb wave tomography imaging with the rapid algorithm for hole and crack detection. Sensors.

[11] Qing X., Li W., Wang Y., Sun H. (2019). Piezoelectric Transducer-Based Structural Health Monitoring for Aircraft Applications. Sensors.

[12] Usama M., Qadir J., Raza A., Arif H., Yau K.L.A., Elkhatib Y., Hussain A., Al-Fuqaha A. (2019). Unsupervised Machine Learning for Networking: Techniques, Applications and Research Challenges. IEEE Access.

[13] Johnson J., Yadav A. (2016). Fault detection and classification technique for HVDC transmission lines using KNN. Information and Communication Technology for Sustainable Development.

[14] Vitola J., Tibaduiza D., Anaya M., Pozo F. Structural Damage detection and classification based on Machine learning algorithms. Proceedings of the 8th European Workshop on Structural Health Monitoring.

[15] Tibaduiza Burgos D.A., Gomez Vargas R.C., Pedraza C., Agis D., Pozo F. (2020). Damage identification in structural health monitoring: A brief review from its implementation to the use of data-driven applications. Sensors.

[16] Chapuis B., Sjerve E. (2018). Sensors, Algorithms and Applications for Structural Health Monitoring: IIW Seminar on SHM, 2015.

[17] Tibaduiza D., Anaya M., Forero E., Castro R., Pozo F. (2016). A Sensor Fault Detection Methodology applied to Piezoelectric Active Systems in Structural Health Monitoring Applications. IOP Conference Series: Materials Science and Engineering.

[18] Avci O., Abdeljaber O., Kiranyaz S., Inman D., Mains M.L., Dilworth B.J. (2020). Structural Health Monitoring with Self-Organizing Maps and Artificial Neural Networks. Topics in Modal Analysis & Testing.

[19] Tibaduiza D.A., Torres-Arredondo M.A., Mujica L., Rodellar J., Fritzen C.P. (2013). A study of two unsupervised data driven statistical methodologies for detecting and classifying damages in structural health monitoring. Mech. Syst. Signal Process..

[20] Sharma V. (2000). Survey of Classification Algorithms and Various Model Selection Methods. J. Mach. Learn. Res..

[21] Alamdari M.M., Rakotoarivelo T., Khoa N.L.D. (2017). A spectral-based clustering for structural health monitoring of the Sydney Harbour Bridge. Mech. Syst. Signal Process..

[22] Torres-Arredondo M.A., Tibaduiza D.A., McGugan M., Toftegaard H., Borum K.K., Mujica L.E., Rodellar J., Fritzen C.P. (2013). Multivariate data-driven modelling and pattern recognition for damage detection and identification for acoustic emission and acousto-ultrasonics. Smart Mater. Struct..

[23] Avci O., Abdeljaber O., Kiranyaz S., Hussein M., Gabbouj M., Inman D.J. (2021). A review of vibration-based damage detection in civil structures: From traditional methods to Machine Learning and Deep Learning applications. Mech. Syst. Signal Process..

[24] Zhang J., Hou Z. (2014). Application of Artificial Immune System in Structural Health Monitoring. J. Struct..

[25] Xiao W. (2012). Structural Health Monitoring and Fault Diagnosis based on Artificial Immune System. Ph.D. Thesis.

[26] Anaya M., Tibaduiza D.A., Pozo F. (2017). Detection and classification of structural changes using artificial immune systems and fuzzy clustering. Int. J. Bio-Inspired Comput..

[27] Shi A. (2015). Structural Damage Assessment Using Artificial Neural Networks and Artificial Immune Systems. Master’s Thesis.

[28] Pozo F., Tibaduiza D.A., Anaya M., Vitola J. A machine learning methodology for structural damage classification in structural health monitoring. Proceedings of the 8th Conference on Smart Structures and Materials, SMART 2017 and 6th International Conference on Smart Materials and Nanotechnology in Engineering, SMN 2017.

[29] Tibaduiza D., Torres-Arredondo M.Á., Vitola J., Anaya M., Pozo F. (2018). A Damage Classification Approach for Structural Health Monitoring Using Machine Learning. Complexity.

[30] Vitola J., Pozo F., Tibaduiza D.A., Anaya M. (2017). A sensor data fusion system based on k-nearest neighbor pattern classification for structural health monitoring applications. Sensors.

[31] Nick W., Asamene K., Bullock G., Esterline A., Sundaresan M. (2015). A Study of Machine Learning Techniques for Detecting and Classifying Structural Damage. Int. J. Mach. Learn. Comput..

[32] Ng. C. (2014). Application of Bayesian-designed artificial neural networks in Phase II structural health monitoring benchmark studies. Aust. J. Struct..

[33] Lee J., Kim S. (2007). Structural Damage Detection in the Frequency Domain using Neural Networks. J. Intell. Mater. Syst. Struct..

[34] Betti M., Facchini L., Biagini P. (2015). Damage detection on a three-storey steel frame using artificial neural networks and genetic algorithms. Meccanica.

[35] Lee E.W.M., Lam H.F. (2011). Intelligent-based Structural Damage Detection Model. Mech. Adv. Mater. Struct..

[36] Villamizar R., Camacho J., Carrillo Y., Pirela L., Jamshidi M., Kreinovich V., Kacprzyk J. (2014). Automatic Sintonization of SOM Neural Network Using Evolutionary Algorithms: An Application in the SHM Problem. Advance Trends in Soft Computing.

[37] Qiu J., Wu Q., Ding G., Xu Y., Feng S. (2016). A survey of machine learning for big data processing. Eurasip J. Adv. Signal Process..

[38] Kohonen T. (1990). The Self-Organizing Map. Proc. IEEE.

[39] Kohonen T., Hynninen J., Kangas J., Laaksonen J. (1996). SOM PAK: The Self-Organizing Map Program Package.

[40] Freeman J., Skapura D. (1991). Neural Networks—Algorithms, Applications, and Programming Techniques.

[41] Graupe D. (2013). Counter Propagation. Principles of Artificial Neural Networks.

[42] Melssen W., Wehrens R., Buydens L. (2006). Supervised Kohonen networks for classification problems. Chemom. Intell. Lab. Syst..

[43] Buethe I., Kraemer P., Fritzen C.P. (2012). Applications of self-organizing maps in structural health monitoring. Key Eng. Mater..

[44] Junior P.O., Conte S., D’Addona D.M., Aguiar P., Bapstista F. (2020). An improved impedance-based damage classification using self-organizing maps. Procedia CIRP.

[45] Zeinali Y., Story B. (2016). Structural Impairment Detection Using Deep Counter Propagation Neural Networks. Procedia Eng..

[46] Jiang S.F., Fu C., Zhang C.M., Wu Z. (2013). A Revised Counter-Propagation Network Model Integrating Rough Set for Structural Damage Detection. Int. J. Distrib. Sens. Netw..

[47] Tibaduiza D.A., Mujica L.E., Rodellar J. (2013). Damage classification in structural health monitoring using principal component analysis and self-organizing maps. Struct. Control Health Monit..

[48] Vitola J., Pozo F., Tibaduiza D.A., Anaya M. (2017). Distributed piezoelectric sensor system for damage identification in structures subjected to temperature changes. Sensors.

[49] Baptista F.G., Filho J.V. (2010). Transducer loading effect on the performance of PZT-based SHM systems. IEEE Trans. Ultrason. Ferroelectr. Freq. Control.

[50] Andhale Y.S., Masurkar F.A., Yelve N.P. (2019). Localization of damages in plain and riveted aluminium specimens using lamb waves. Int. J. Acoust. Vib..

[51] Bermes C. (2006). Generation and Detection of Nonlinear Lamb Waves for the Characterization of Material Nonlinearities. Ph.D. Thesis.

[52] National Instruments (2021) What Is LabView?. https://www.ni.com/es-co/shop/labview.html.

[53] Rostami J., Chen J., Tse P.W. (2017). A Signal Processing Approach with a Smooth Empirical Mode Decomposition to Reveal Hidden Trace of Corrosion in Highly Contaminated Guided Wave Signals for Concrete-Covered Pipes. Sensors.

[54] Oshana R., Oshana R. (2006). 4—Overview of Digital Signal Processing Algorithms. DSP Software Development Techniques for Embedded and Real-Time Systems.

[55] Banerjee M.B., Chatterjee T.N., Roy R.B., Tudu B., Bandyopadhyay R., Bhattacharyya N. (2017). Multivariate preprocessing techniques towards optimising response of fused sensor from electronic nose and electronic tongue. Proceedings of the 2016 International Conference on Computing, Communication and Automation (ICCCA).

[56] Palit M., Tudu B., Bhattacharyya N., Dutta A., Dutta P.K., Jana A., Bandyopadhyay R., Chatterjee A. (2010). Comparison of multivariate preprocessing techniques as applied to electronic tongue based pattern classification for black tea. Anal. Chim. Acta.

[57] Jolliffe I. (2013). Principal Component Analysis.

[58] Mujica L.E., Rodellar J., Fernández A., Güemes A. (2011). Q-statistic and t2-statistic pca-based measures for damage assessment in structures. Struct. Health Monit..

[59] Taşdemir K., Laaksonen J., Honkela T. (2011). Spectral Clustering as an Automated SOM Segmentation Tool. Advances in Self-Organizing Maps.

[60] Barletta V., Caivano D., Nannavecchia A., Scalera M. (2020). A Kohonen SOM Architecture for Intrusion Detection on In-Vehicle Communication Networks. Appl. Sci..

[61] Ullah A., Haydarov K., Ul Haq I., Muhammad K., Rho S., Lee M., Baik S.W. (2020). Deep Learning Assisted Buildings Energy Consumption Profiling Using Smart Meter Data. Sensors.

[62] Milano Chemometrics and QSAR Research Group (2021) Kohonen and CPANN Toolbox (for Matlab). https://michem.unimib.it/download/matlab-toolboxes/kohonen-and-cpann-toolbox-for-matlab/.

[63] Ballabio D., Consonni V., Todeschini R. (2009). The Kohonen and CP-ANN toolbox: A collection of MATLAB modules for self organizing maps and counterpropagation artificial neural networks. Chemom. Intell. Lab. Syst..

[64] Ballabio D., Vasighi M. (2012). A MATLAB toolbox for Self Organizing Maps and supervised neural network learning strategies. Chemom. Intell. Lab. Syst..

[65] Ballabio D., Consonni V. (2013). Classification tools in chemistry. Part 1: Linear models. PLS-DA. Anal. Methods.

